# Mild *RPE65*-Associated Inherited Retinal Dystrophies: A Multimodal Clinical and Genetic Evaluation

**DOI:** 10.1167/tvst.14.11.24

**Published:** 2025-11-18

**Authors:** Lasse Wolfram, David A. Merle, Laura Kühlewein, Melanie Kempf, Krunoslav Stingl, Susanne Kohl, Katarina Stingl

**Affiliations:** 1 Department for Ophthalmology, University Eye Clinic, Eberhard Karls University of Tübingen, Tübingen, Germany; 2 Department for Ophthalmology, Institute for Ophthalmic Research, Eberhard Karls University of Tübingen, Tübingen, Germany; 3 Center for Rare Eye Diseases, Eberhard Karls University of Tübingen, Tübingen, Germany

**Keywords:** inherited retinal diseases (IRDs), RPE65, leber congenital amaurosis (LCA), retinitis pigmentosa (RP), congenital stationary night blindness (CSNB), fundus albipunctatus (FA), gene therapy, voretigene neparvovec, dark-adapted perimetry

## Abstract

**Purpose:**

RPE65 is a key enzyme in the visual cycle, converting all-*trans* retinyl esters into 11-*cis* retinol, a crucial step in regenerating the photopigment necessary for vision. Mutations cause a spectrum of inherited retinal diseases (IRDs), from severe generalized early-onset dystrophies, such as Leber congenital amaurosis, to classical retinitis pigmentosa or mild phenotypes, including congenital stationary night blindness, such as fundus albipunctatus.

**Methods:**

We analyzed two independent patients with mild *RPE65*-associated IRDs using multimodal diagnostics, including best-corrected visual acuity; Goldman visual field; dark-adapted testing, including scotopic perimetry; full-field electroretinography; and multimodal retinal imaging. Phenotypes were evaluated based on existing literature and predicted variant impact.

**Results:**

Both patients exhibited overall mild IRDs with only slightly impaired rod function and largely preserved cone function. Identified *RPE65* missense variants likely allow partial enzyme function, consistent with comparatively mild and slowly progressive disease. Superior rod scotomas and mid-peripheral morphologic changes were identified despite normal or near-normal full-field function.

**Conclusions:**

Functional rod changes in the inferior mid-periphery of the retina, which may be followed by metabolic stress and structural retinal changes, seem to be the hallmark of mild *RPE65*-associated IRDs and may represent early site-specific pathology. These changes may be linked to an increased susceptibility to UV-induced retinal damage associated with *RPE65* mutations. Local rod function assessment is critical for proper disease monitoring and guiding therapeutic decisions.

**Translational Relevance:**

Localized multimodal diagnostics help detect early changes in mild *RPE65*-associated IRDs, supporting precise monitoring and gene therapy counseling.

## Introduction

Retinitis pigmentosa (RP) is a group of inherited retinal diseases (IRDs) characterized by progressive photoreceptor degeneration, typically leading to night blindness, visual field constriction, and eventual loss of central vision.^1^ Among the various genetic variants associated with RP, those in the *RPE65* gene are known to typically cause a particularly severe form of the disease, often presenting with early onset and rapid progression, as in Leber congenital amaurosis (LCA; LCA2).^2^ This can be attributed to the RPE65 protein's role as an isomerohydrolase, responsible for converting all-*trans* retinyl ester into 11-*cis* retinol within the retinal pigment epithelium, a crucial step in the regeneration of visual pigments.^3^ *RPE65*-related IRDs have gained clinical relevance following the approval of voretigene neparvovec (Luxturna) in 2017/2018.^4^

Recent advancements in genetic testing and a deeper understanding of IRD phenotypes have identified a subset of patients with *RPE65*-associated IRD who experience a significantly milder disease course, even in advanced age.^5^ The mildest phenotype of this spectrum is fundus albipunctatus (FA), one of the clinical forms of congenital stationary night blindness (CSNB).^6^^–^^9^ Clinical features in FA are night blindness with delayed dark adaptation and typical subtle fundoscopic changes with white dots in an overall nonprogressive disease course, having been first reported by Lauber in 1910.^10^ In some cases, however, FA is associated with cone dystrophy,^11^^,^^12^ and in fact, the spectrum of *RPE65*-related phenotypes with white fundus lesions may also include more generalized presentations of retinal dystrophy,^8^ resembling the more typical *RPE65*-related retinal dystrophy.

The typical genotype associated with FA is the autosomal recessive *RDH5*-related retinal dystrophy. *RDH5* encodes the retinol dehydrogenase 5 protein, which is an important factor in the rhodopsin regeneration cycle. Its reduction results in impaired regeneration of rhodopsin, leading to prolonged dark adaptation and night blindness. Notably, recent studies have also reported maculopathy in a subset of patients with *RDH5*-related FA, suggesting that even within classically stationary phenotypes, structural central retinal involvement may occur.^13^^–^^15^ In recent years, FA has also been genetically attributed to mutations in the *RLBP1* gene, as well as *RPE65*.^6^^,^^7^^,^^16^ In addition to these, biallelic variants in other visual cycle genes such as *LRAT* and *RDH8* have also been associated with FA-like phenotypes, often presenting with white or yellow dot-like retinal lesions, further expanding the genetic differential diagnosis of such findings.^17^^–^^19^

So far, these cases have been presented with typical clinical retinal diagnostics using visual acuity, electroretinography, and retinal imaging (fundoscopy, optical coherence tomography [OCT], and fundus autofluorescence [FAF] imaging). In this study, we present two cases of mild *RPE65*-associated IRDs, using detailed multimodal retinal diagnostics, including the retinotopic evaluation of rod sensitivity using dark-adapted chromatic perimetry (DACP), as well as metabolic and oximetric retinal imaging to enhance the understanding of the retinal physiology changes present in this phenotype.

A multimodal approach is essential for understanding the retinal physiology underlying this hypomorphic phenotype, given the availability of voretigene neparvovec (Luxturna), which offers promising therapeutic options but requires timely and accurate diagnosis to optimize patient outcomes.^20^ Whereas even mild *RPE65*-associated phenotypes showing clinical progression do benefit from the treatment,^21^ completely nonprogressive phenotypes might sustain their day vision well preserved until late age without the risks of surgical treatment. Therefore, detailed multimodal retinal diagnostics beyond the usual routine standards might be necessary for an optimal consulting and risk-benefit evaluation for patients with mild *RPE65*-associated IRDs.

## Methods

### Patient Selection

Two patients who initially presented at the University Eye Hospital in Tübingen (Germany) with comparatively mild symptoms, primarily night vision difficulties, and were later found to carry biallelic *RPE65* variants, were included for a multimodal assessment of the retinal physiology.

### Ophthalmologic Examination

Clinical examination included best-corrected visual acuity (BCVA); slit-lamp biomicroscopy and dilated fundoscopy; pseudocolor fundus photography (California P200DTx, Optos, Dunfermline, UK); FAF imaging (California P200DTx; Optos, Dunfermline, UK); OCT (Spectralis; Heidelberg Engineering, Heidelberg, Germany); 90° kinetic perimetry (Goldmann visual field, GVF; Octopus 900, Haag-Streit Diagnostics, Haag-Streit, Wedel, Germany); DACP using cyan stimuli (Medmont, Nunawading, Victoria, Australia; using a 30° raster with normal dark-adapted values up to 60 dB^22^); color perception (Panel D-15 color cups); custom-built chromatic pupil campimetry (CPC) with scotopic and photopic protocols^23^^,^^24^; full-field electroretinography (ffERG) measuring dark-adapted (DA) and light-adapted (LA) responses following the International Society for Clinical Electrophysiology of Vision standards; full-field stimulus threshold testing (FST) measuring dark-adapted thresholds using white, blue, and red stimuli with 0 dB calibrated to 0.01 cd⋅s/m^2^ (Espion 2 and Espion 3, Diagnosys, Lowell, MA, USA) with all dark-adapted examinations performed after 20 minutes of dark adaptation (control values in healthy adults for white [−54.9 ± 6.6 dB], blue [−60.9 ± 5.6 dB], and red [−36.8 ± 6.7 dB] stimuli); retinal oximetry (Oxymap; T1 Retinal Oximeter, Oxymap, Reykjavik, Iceland); and flavoprotein fluorescence (FPF; Ocumet Beacon, Ocusciences, Ann Arbor, MI, USA). The readout value of the FPF score is age-dependent,^25^ with normal values for ages 20 to 30 years, ranging from 20 to 40 and increasing to 100 to 120 in the ninth decade of life.

### Genetic Testing

Molecular diagnostic testing was conducted via an in silico panel for genes known to be associated with IRDs, based on whole-genome sequencing,^26^ and subsequent segregation analysis was performed by polymerase chain reaction and Sanger sequencing in both parents of the respective patient. Variants were classified based on the American College of Medical Genetics and Genomics guidelines by the Institute of Medical Genetics and Applied Genomics, University of Tübingen, Tübingen, Germany.

### Ethics Approval and Consent to Participate

This study, involving human participants, was approved by the Ethics Committee of the University of Tübingen (367/2019BO1, 116/2015BO2, 637/2017BO1) and adhered to the principles of the Declaration of Helsinki. Written informed consent for genetic testing was obtained for all individuals.

### Consent for Publication

All individuals (and their legal guardians, where applicable), whose personal data are included in this article, gave written informed consent for their personal or clinical details, along with any identifying images, to be published in this study.

### Availability of Data and Materials

Raw data from whole-genome sequencing are not publicly available to protect individuals’ privacy, in compliance with the European General Data Protection Regulation. Access to the data may be granted upon reasonable request and subject to appropriate ethical and legal approvals.

## Results

### Patient SRP1409

**Figure 1.**
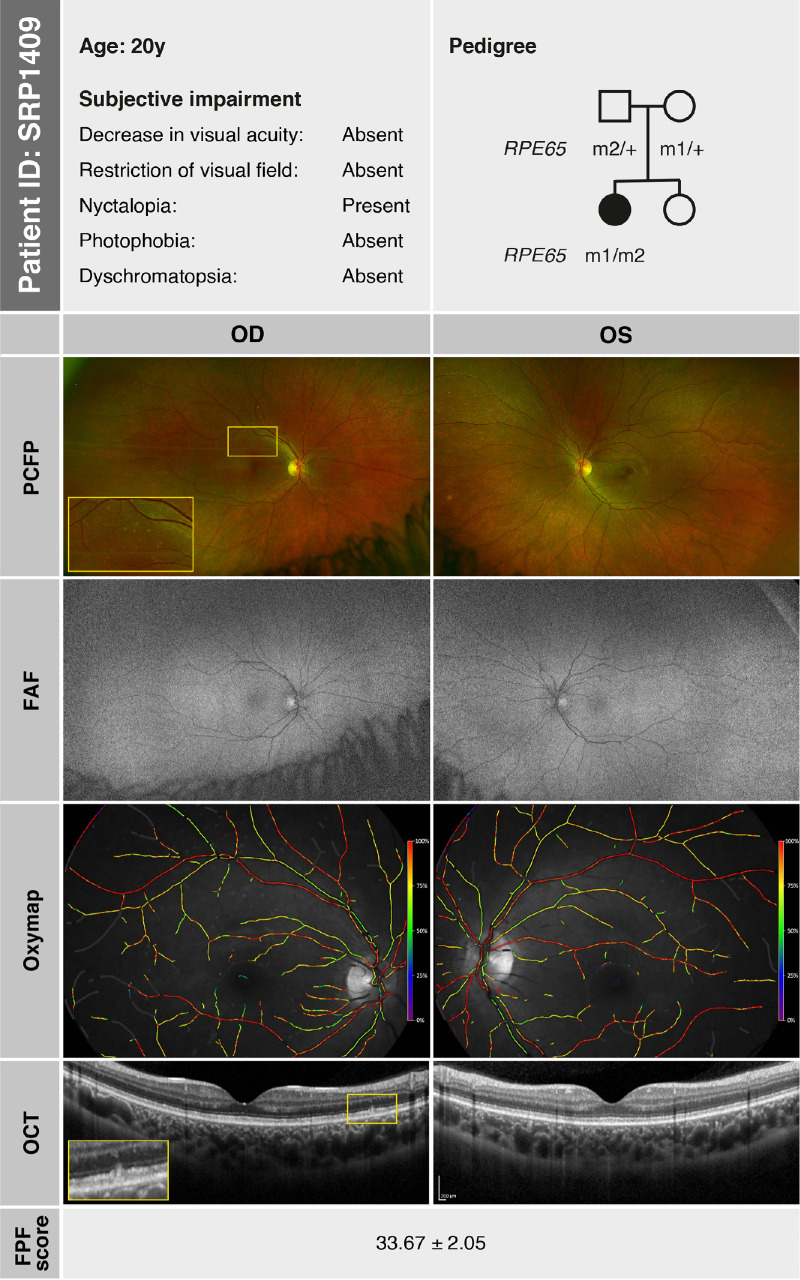
SRP1409 (at age 20 years): summary of subjective symptoms (based on patient history), pedigree analysis, and morphologic findings—including pseudocolor fundus photography (PCFP), FAF imaging, and OCT—as well as functional assessments using retinal oximetry (Oxymap) and FPF score. The whitish albipunctatus lesion seen in the PCFP (*inset*) corresponds to a focal disturbance in the space between the ELM and the RPE on OCT (*inset*). The identified mutations are represented as follows: m1, *RPE65* c.718G>T, p.(Val240Phe); m2, *RPE65* c.1022T>C, p.(Leu341Ser).

SRP1409, a female, first presented to our clinic at the age of 6 years, reporting impaired night vision since the age of 3 years with no difficulties with daytime vision. The family history was devoid of IRDs, and there was no indication of consanguinity. At the time of consultation, her BCVA was 20/32 in both eyes (OD: +3.00 DS/−3.25 DC × 25°; OS: +3.75 DS/−3.00 DC × 165°). Anterior segment examination was unremarkable. Fundus examination revealed small whitish lesions in the mid-periphery of both eyes, with a configuration similar to FA. GVF demonstrated intact outer boundaries without defects. Saturated and desaturated Panel D-15 testing revealed physiologic results. ffERG indicated severely reduced responses under DA conditions with nondetectable DA 0.01 and reduced DA 3.0, while LA responses were near normal, with LA 3.0 flash responses slightly below normal limits and LA 3.0 flicker responses within normal limits. At a follow-up at age 20 years, SRP1409 reported a subjective worsening of night blindness. Her BCVA was 20/20 in both eyes. Anterior and posterior segment examinations were as at the initial visit. FAF imaging revealed an overall reduced autofluorescence signal in both eyes. On OCT, the subtle deposits visualized in the mid-periphery spanned the region between the external limiting membrane (ELM) and the retinal pigment epithelium (RPE) (Fig. 1, inset). GVF remained fully intact. ffERG continued to show severely reduced DA responses, with LA responses preserved but slightly diminished. FST for the right eye revealed rod-mediated dark adaptation thresholds that were only mildly elevated for white (−48.0 dB), blue (−47.5 dB), and red (−29.6 dB) light. DACP revealed superior dark-adapted rod sensitivity defects corresponding to structural abnormalities in the region of the lower arcade. The photopic CPC showed normal pupil responses to cone-specific stimuli, indicating a well-preserved cone number. This finding is consistent with the normal photopic electroretinography (ERG) amplitudes, reflecting near-normal cone function. The scotopic CPC, however, showed an overall reduction of pupil responses to rod stimuli compared to physiological values,^22^^,^^23^ most pronounced in the center and inferior retinal mid-periphery, indicating local rod cell number loss, again in concordance with the scotopic ERG. Retinal oximetry revealed overall well-preserved blood flow and normal retinal oxygenation in the arterial and venous system.^27^^,^^28^ A color-coded map of relative hemoglobin oxygen saturation (%) in both eyes is shown in Figure 1, illustrating normal oxygenation levels in the arterial system (close to 100%) and in the venous system (approximately 60%). FPF of regions around the optic nerve, the macula, and temporal to the macula in both eyes were measured at 33.67 ± 2.05, indicating physiologic values of mitochondrial flavoprotein.^25^

Genetic testing revealed a compound-heterozygous constellation of the maternally inherited likely pathogenic missense variant c.718G>T, p.(Val240Phe) and the paternally inherited pathogenic missense variant c.1022T>C, p.(Leu341Ser) in the *RPE65* gene. Based on detailed consulting, including the potential use of voretigene neparvovec (Luxturna) and its benefit-risk ratio, the patient opted for a watchful waiting approach given the relatively stable *RPE65* phenotype with overall still preserved rod dark-adapted sensitivity.

**Figure 2.**
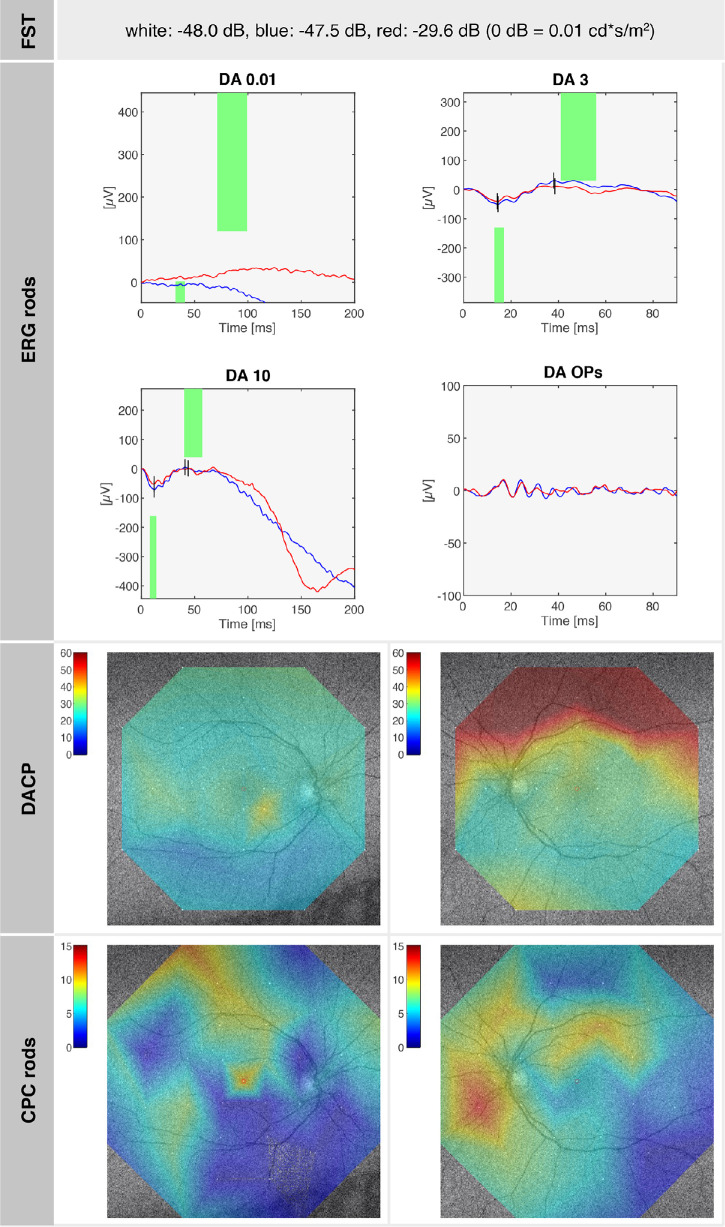
SRP1409 (at age 20 years): summary of rod function–related test results, with close-to-normal FST values, reduced rod ERG responses as well as DACP, and rod function assessed by CPC, demonstrating rod cell loss and rod sensitivity loss along the retinal lower mid-periphery. A normal dark-adapted sensitivity in a healthy eye is 60 dB in the entire tested field, and a normal relative pupil response on rod-tailored stimuli is 13% ± 6% in the center. The superior rod scotomas are overlaid correspondingly on the fundus image, correlating with the inferior retinal mid-periphery.

**Figure 3.**
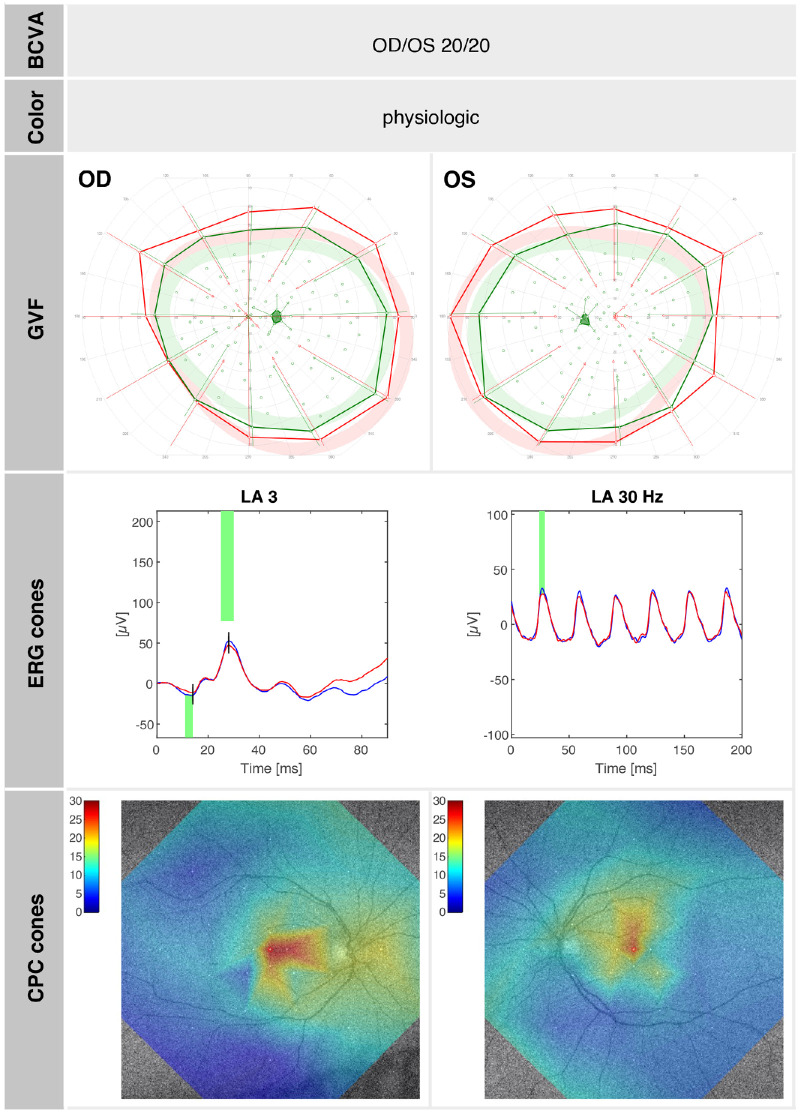
SRP1409 (at age 20 years): summary of cone function–related test results showing normal cone functionality with BCVA, color perception, GVF, cone ERG responses, and cone function assessed by CPC. A normal relative pupil response on cone-tailored stimuli is 20% ± 5% in the center.

A summary of the morphologic and functional findings of SRP1409 at age 20 years is presented in Figures 1 to 3. Figure 1 illustrates the pedigree and morphologic characteristics, while Figures 2, 3 depict cone function and rod function, respectively.

### Patient SRP1465

**Figure 4.**
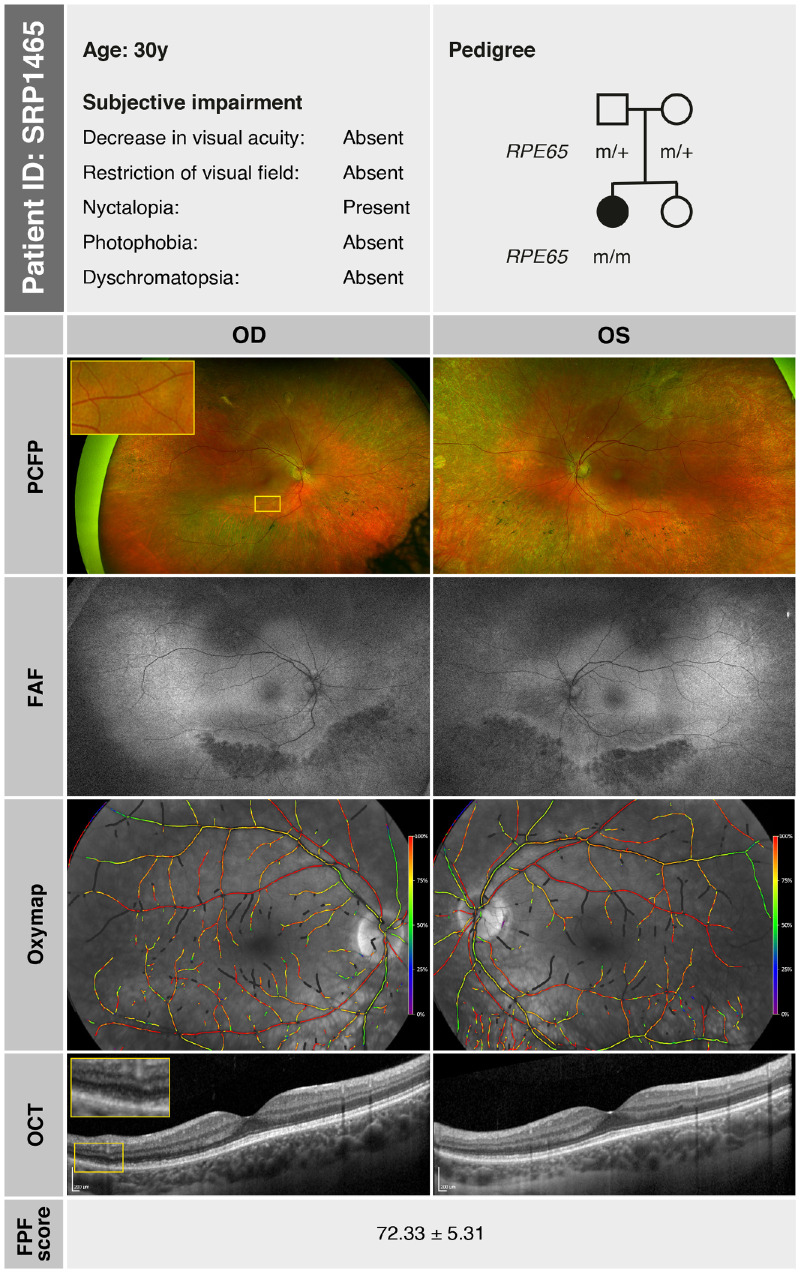
SRP1465 (at age 30 years): summary of subjective symptoms (based on patient history), pedigree analysis, and morphologic findings—including PCFP, FAF imaging, and OCT—as well as functional assessments using retinal oximetry (Oxymap) and FPF score. The diffuse atrophic area visible in the PCFP (*inset*) corresponds to a disturbance in the space between the ELM and the RPE with ellipsoid zone loss and RPE disruption on OCT (*inset*). The identified mutation is represented as follows: m, *RPE65* c.65T>C, p.(Leu22Pro).

Patient SRP1465, also a female, presented to our clinic at the age of 30 years, reporting difficulties with night vision since childhood. The family history was negative for IRDs, and there was no known consanguinity. Clinical examination revealed full visual acuity (OD: +2.50 DS/−3.50 DC × 175°; OS: +2.00 DS/−2.75 DC × 180°), an unremarkable anterior segment, and small whitish and yellowish dots, as well as pigmented lesions and atrophic circular lesions in the retinal mid-periphery. FAF imaging showed a slightly reduced overall autofluorescence with localized hypoautofluorescence in the inferior and superior mid-periphery, indicating early outer retinal alterations. OCT detected photoreceptor atrophy, beginning at the inferior temporal arcade, with a well-preserved central macula. In the area of beginning atrophy, subtle deposits were also visible on OCT and appeared to span the space between the ELM and the RPE (Fig. 4, inset). GVF testing revealed physiological outer boundaries, although with a few peripheral scotomas superiorly with the Goldmann III4e stimulus and slight concentric narrowing of the outer boundaries with the Goldmann I4e stimulus. DACP showed superior defects corresponding to damage in the region of the lower arcade, while the remaining retina revealed good dark-adapted sensitivity. Panel D-15 testing revealed physiologic results. ffERG demonstrated moderately reduced scotopic responses (notably DA 0.01), whereas photopic single-flash (LA SF) responses were only slightly attenuated. FST testing of the right eye indicated a rod-mediated, near-normal dark adaptation threshold for blue (−55.5 dB) and red (−30.1 dB) light. CPC revealed overall reductions compared to physiological values under not only scotopic but also photopic conditions.^22^^,^^23^ Retinal oximetry demonstrated overall well-preserved blood flow and normal oxygenation levels in both the arterial and venous retinal vasculature.^27^^,^^28^ Figure 4 shows a color-coded map of relative hemoglobin oxygen saturation (%) in both eyes, with arterial saturation near 100% and venous saturation around 60%. The FPF of regions around the optic nerve, the macula, and temporal to the macula in both eyes was measured at 72.33 ± 5.31, indicating significantly increased metabolic stress levels.

SRP1465 carried the homozygous likely pathogenic missense variant c.65T>C, p.(Leu22Pro) in the *RPE65* gene, and segregation analysis confirmed that both parents were heterozygous carriers of this variant. Thorough counseling was provided regarding the diagnosis, possible disease progression, and therapeutic options, including the potential use of voretigene neparvovec (Luxturna). This included a detailed discussion of the benefits and potential side effects. Due to very mild findings with barely any impact on daily vision and overall well-preserved rod-mediated dark adaptation, we decided, together with the patient, not to treat at this time point. Further follow-ups are needed to evaluate the progression of the phenotype and thus possible future benefit from potential gene therapy with voretigene neparvovec (Luxturna).

**Figure 5.**
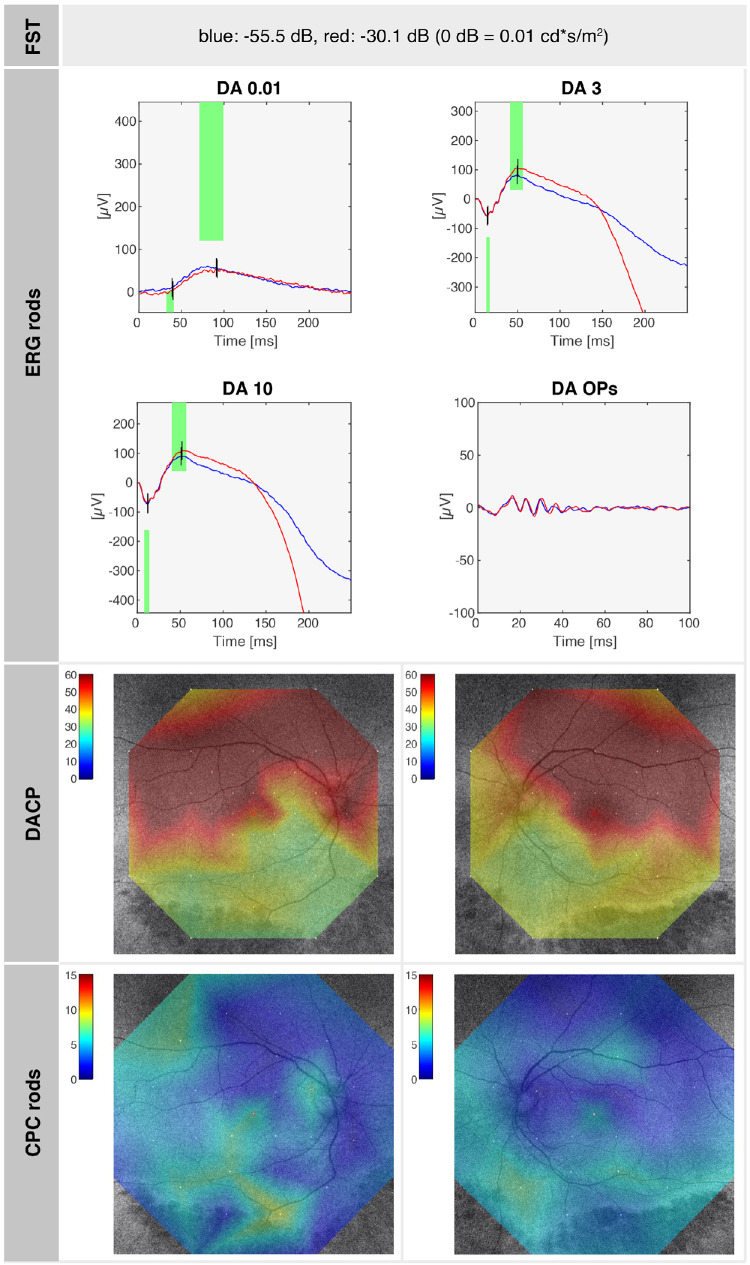
SRP1465 (at age 30 years): summary of rod function–related test results with normal FST values, slightly reduced rod ERG responses, and DACP: the superior rod scotomas are overlaid correspondingly on the fundus image correlating with the inferior retinal mid-periphery. A normal dark-adapted sensitivity in a healthy eye is 60 dB in the entire tested field, and a normal relative pupil response on rod-tailored stimuli is 13% ± 6% in the center. The rod function assessed by CPC shows overall reduced pupil response on rod stimuli, indicating a reduced rod cell number.

**Figure 6.**
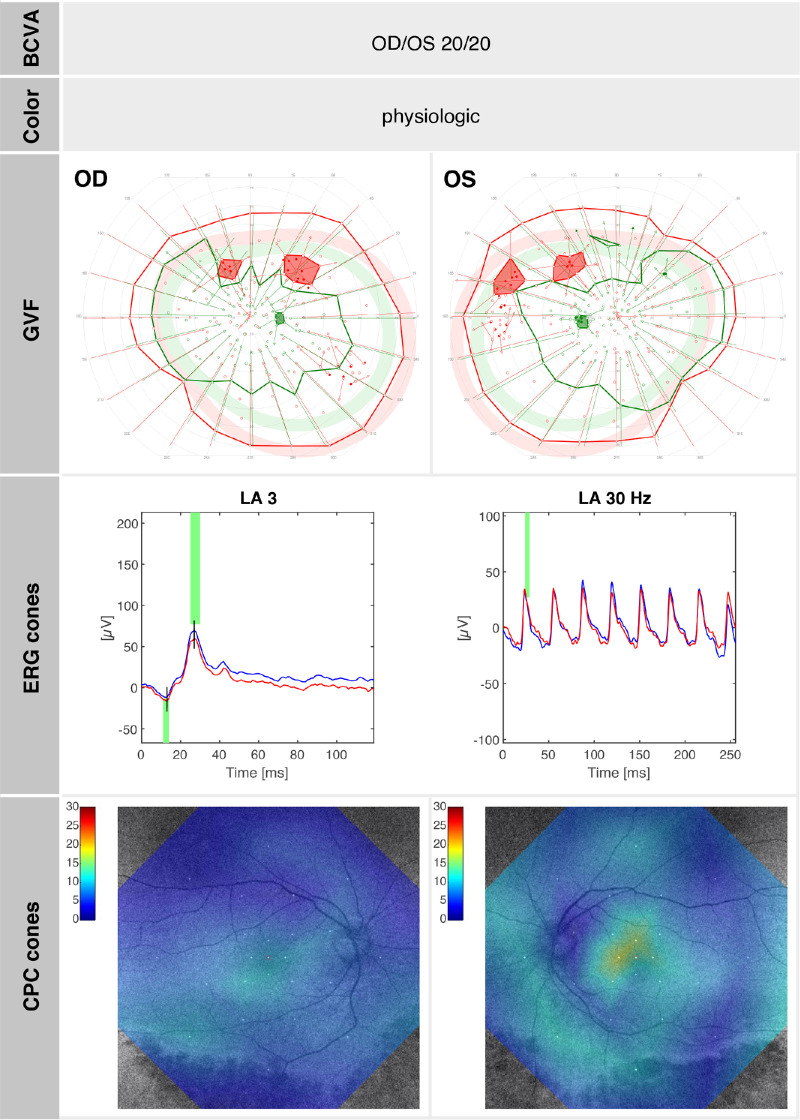
SRP1465 (at age 30 years): summary of cone function–related test results with normal BCVA, color perception, and GVF with only small superior scotomas. The cone ERG responses and cone function assessed by CPC are both slightly reduced, indicating a slightly reduced cone cell number. A normal relative pupil response on cone-tailored stimuli is 20% ± 5% in the center.

A summary of the morphologic and functional findings of SRP1465 at age 30 years is presented in Figures 4 to 6. Figure 4 illustrates the pedigree and morphologic characteristics, while Figures 5, 6 depict cone function and rod function, respectively.

## Discussion

The two cases of *RPE65*-associated IRDs presented here exhibit characteristic features of the milder end of the *RPE65* disease spectrum. While SRP1409 fulfills the clinical criteria of classical FA with preserved but functionally impaired rod function and normal or near-normal cone function, SRP1465 demonstrates a mild or hypomorphic *RPE65*-associated phenotype that also exhibits pigmentary changes and detectable DA 0.01 responses. Both patients had completely normal or near-normal day vision without subjective symptoms in the photopic range. In concordance with FA, an autosomal recessive subtype of CSNB,^7^ SRP1409’s only symptom was disturbed night vision. This phenotype represents the mildest spectrum of *RPE65*-related retinal disease. In contrast, SRP1465 showed localized outer retinal atrophy and mild cone dysfunction, indicating a potentially slowly progressive disease course.

Our findings in these two cases demonstrate that, despite minimal symptoms, retinal physiology likely begins to show rod dysfunction in the inferior mid-periphery. In SRP1465, this is accompanied by localized functional deterioration detectable on FAF imaging. A potential correlation with cumulative sunlight exposure in these inferior regions may play a role. This observation is particularly noteworthy, as such changes are not detectable by routine diagnostics and require dark-adapted perimetry for identification. The significance lies not only in assessing early functional progression but also in providing guidance on sunlight protection strategies for patients.

Although both patients were young adults, no clinically relevant progression has been detected so far in either case. These aspects led us to the diagnosis of CSNB in SRP1409. The presence of characteristic whitish lesions in SRP1409 and predominantly pigmentary but only subtle whitish lesions in SRP1465 further supports the interpretation that SRP1409 represents classical FA, while SRP1465 reflects a hypomorphic phenotype within the broader *RPE65* disease spectrum.

The presence of such phenotypic variability, even among patients with biallelic *RPE65* variants, underscores the potential influence of both genetic background and metabolic stress on disease progression. These findings support the notion that *RPE65*-related disease encompasses a continuum, ranging from nonprogressive FA-like forms to slowly progressive hypomorphic phenotypes. Moreover, they highlight the potential utility of FPF as a sensitive biomarker for detecting early subclinical retinal changes in this context.

Although night blindness was the only reported symptom in both cases, each patient exhibited a clear relative rod scotoma in the superior visual field. In SRP1465, this scotoma corresponded to early signs of outer retinal atrophy, suggesting a mildly progressive phenotype compared to SRP1409, who showed only localized functional impairment without morphologic degeneration. This rod dysfunction in the superior visual field, retinotopically mapping to the inferior mid-periphery, may represent an early and possibly characteristic feature of mild *RPE65*-associated retinal dystrophies, which can be missed if only ffERG and FST are tested. We hypothesize that these superiorly located scotomas may arise from cumulative environmental stress, particularly light exposure. As previously described in *RHO*-associated disease,^29^ chronic UV exposure—predominantly affecting the upper visual field—can disrupt rhodopsin regeneration, impair rod metabolism, and contribute to photoreceptor degeneration.

It has previously been shown that genetic variations in *RPE65* can influence retinal resistance to light-induced degeneration by affecting rhodopsin regeneration.^30^ This highlights the complex interplay between genetic mutations and environmental factors, such as light exposure, in the progression of retinal dystrophies. The superior rod scotomas observed in the patients may result from a combination of genetic mutations in the *RPE65* gene affecting rod function and environmental factors like UV exposure, leading to retinal degeneration in specific areas of the visual field.

FPF serves as a biomarker of mitochondrial dysfunction and oxidative stress in the RPE.^25^ Elevated FPF values indicate an accelerated metabolic imbalance, with the higher FPF in SRP1465 suggesting greater mitochondrial dysfunction, preceding morphologic degeneration. This may be associated with increased disease severity or environmental factors such as prolonged UV exposure due to older age. These findings support the role of oxidative stress in the progression of *RPE65*-associated retinal dystrophies, highlighting FPF as a potential marker for disease status and progression. Such information is clinically relevant, given the possibility of gene therapy with voretigene neparvovec (Luxturna) and consulting the patient about the possible progression of the disease, even if there is barely any benefit of gene therapy in the current stage.

Retinal oximetry revealed well-preserved blood flow and oxygenation in both cases, consistent with their overall structural integrity, compared with advanced cases of RP.^31^

These two mild cases of *RPE65*-associated IRDs underscore the diagnostic challenges in differentiating stationary, FA-like phenotypes from slowly progressive, hypomorphic forms. Our findings illustrate that biallelic *RPE65* variants can result in distinctly different clinical presentations, depending on their impact on protein function. Understanding how specific *RPE65* variants contribute to this phenotypic diversity—from nonprogressive FA to classical RP or LCA—offers valuable insights into the underlying mechanisms of disease expression and progression.

The three missense variants identified in our patients have all been previously reported in the literature; notably, none of them are located within the known active sites of *RPE65*,^32^ supporting their likely residual enzymatic activity and association with milder phenotypes.

The missense variant c.65C>T, p.(Leu22Pro) has repeatedly been reported in the literature. The variant was initially reported in compound heterozygosity with p.(His68Tyr) in a patient with RP who had early-onset nyctalopia as well as macular involvement, leading to severely reduced BCVA,^33^^,^^34^ probably due to the contribution of the counter missense allele. Functional testing using an isomerase activity assay demonstrated that the p.(Leu22Pro) variant results in 13.5% of wild-type enzymatic activity. Clinically, the residual activity is associated with a comparatively mild phenotype, characterized by early-onset nyctalopia and diffuse irregular pigmentation, but with overall relatively preserved cone function, including BCVA, as observed in SRP1465.^35^ Li and coworkers showed that 26 S proteasome non-ATPase regulatory subunit 13 (PSMD13) is a negative regulator of RPE65, playing a critical role in regulating the pathogenicity of certain mutations, including p.(Leu22Pro), by mediating rapid degradation of mutant RPE65 via a ubiquitination- and proteasome-dependent nonlysosomal pathway. RPE65-p.(Leu22Pro) is misfolded and forms aggregates. Interaction of PSMD13 with mutant RPE65 promoted degradation of misfolded but not properly folded mutant RPE65.^36^^,^^37^ Voretigene neparvovec (Luxturna) treatment in a 5-year-old female patient was reported in the literature, with the very same homozygous c.65T>C;p.(Leu22Pro) genotype but a more severe phenotype than observed in SRP1465. Similar to some pediatric patients showing an inflammatory reaction after gene therapy, she developed subretinal deposits in the macula, mild vitreous opacities, and a large area of subretinal whitening inferiorly.^38^ Fortunately, these findings gradually resolved in both eyes, leading to an overall improvement in both BCVA and nyctalopia after gene therapy.

The missense variant c.718G>T, p.(Val240Phe) has been described only once in the literature, in two Costa Rican brothers with early-onset retinal dystrophy. While both presented with nyctalopia from early childhood, their overall phenotype was comparatively mild, with visual acuity remaining relatively well preserved at least through adolescence, aligning with the clinical picture of SRP1409.^39^ In contrast, the missense variant c.1022T>C; p.(Leu341Ser) has been recurrently reported in the literature in both LCA and other *RPE65*-related IRDs, demonstrating a wide spectrum of functional outcomes. Sallum and colleagues^40^ described seven individuals from five families with LCA or early-onset retinal dystrophy, all with early disease onset and markedly reduced BCVA. Conversely, Lopez-Rodriguez et al.^41^ reported a compound heterozygous case carrying the missense variants p.(Leu341Ser) and p.(Arg515Trp), showing fundus albipunctatus–like changes and comparatively preserved BCVA at age 10 years, more resembling the phenotype observed in SRP1409. Maguire and colleagues^42^ documented a decline in BCVA in a 20-year-old woman with LCA following gene therapy with voretigene neparvovec (Luxturna), underscoring the need for careful, individualized consideration in gene therapy decisions. However, neither of these variants has been functionally tested to date.

Our findings underscore the crucial importance of multimodal clinical diagnostics, including a retinotopically correct understanding of dark-adapted retinal functionality. Only with evaluations such as DACP and CPC,^22^^,^^43^ follow-ups of inferior scotomas are truly possible, as their presence and evolution are not visible with full-field examinations such as ffERG and FST, commonly used by IRD specialists.

The particular importance of such detailed multimodal evaluation is given in the context of an available gene therapy with voretigene neparvovec (Luxturna). The primary target for this gene therapy treatment is rods, which benefit most from the restoration of the RPE65 enzyme in the pigment epithelium and whose dark adaptation can be improved by the treatment.^44^ The efficacy of this treatment is dependent on the availability of the target cells, which leads to the already well-established fact: the earlier the treatment, the better the outcome.^20^ Clinical observations suggest that gene therapy is effective from childhood through young adulthood in *RPE65*-associated retinal dystrophies. However, there may be a critical period—typically during school age or adolescence—when progressive retinal degeneration disrupts retinal energy homeostasis. In some cases, this may result in rapidly developing chorioretinal atrophy following initially successful treatment, likely due to a metabolic imbalance triggered by rod photoreceptor rescue.^45^^,^^46^ This indicates even more the importance of early treatment. Successful treatment has been reported already in children with mild phenotypes of *RPE65* retinal dystrophy.^21^ However, in the case of stationary, rather nonprogressive phenotypes, such as FA, the potential benefit of treatment may be limited—although this remains unproven due to the lack of published data on the use of voretigene neparvovec (Luxturna) in patients with *RPE65*-associated FA-like presentations. The patients have good daylight vision, which could potentially be compromised by adverse events during surgery. A potential impact on rod functionality might be anticipated; however, in our patients, rod functionality measured with FST is only slightly impaired,^47^^,^^48^ suggesting that the actual subjective effect beyond the local rod scotoma may be minimal. A logical option based on the physiological findings reported here would be to treat the inferior retina, which is the most susceptible to sunlight-related damage. However, long-term follow-ups have been chosen in concordance with the patients’ wishes in these cases to evaluate the potential of these phenotypes for progression and the subjective need for therapy.

Hypothetical effects of voretigene neparvovec (Luxturna) on the CSNB-like phenotypes, such as improvement in night blindness, remain to be evaluated. All in all, we endorse careful assessment, including retinal diagnostics, such as DACP and FPF, where available, and comprehensive counseling on the diagnosis, potential outcomes with and without treatment using voretigene neparvovec (Luxturna), and current research evidence regarding potential benefits relative to the disease's current state. Additionally, we recommend taking the patient's daily life limitations into account to thoroughly assess the overall risk-benefit ratio in each individual case. Ultimately, our goal is to facilitate informed shared decision-making, ensuring the best possible medical care.

Our study is not without limitations. First, the sample size is small, primarily due to the extreme rarity of these very mild cases of *RPE65*-related retinal disease. The low prevalence of these cases limits our ability to draw definitive conclusions on a group statistics basis, especially regarding factors to consider when deciding whether to administer voretigene neparvovec (Luxturna). To better identify clinical and genetic indicators for a mild disease course, larger cohorts of patients with mild forms of *RPE65*-associated disease, examined with novel multimodal diagnostics, would be beneficial. Such studies could help refine patient selection for treatment, potentially even at the initial visit before significant disease progression occurs. However, it is important to acknowledge that such cases will likely remain rare. Longitudinal studies would further enhance our understanding of the outcomes of voretigene neparvovec (Luxturna) in patients with mild *RPE65*-associated IRDs.

This study underscores the phenotypic variability of *RPE65*-associated retinal disease and the value of multimodal diagnostics in distinguishing stationary, hypomorphic presentations from progressive forms. In particular, SRP1465 illustrates the importance of recognizing mild phenotypes that may diverge from classical FA both structurally and functionally. Superior rod scotomas and mid-peripheral retinal changes may serve as hallmark features of such phenotypes, potentially influenced by UV exposure and depicting the localization of the earliest retinal alterations. Accurate classification of these variants is essential for appropriate patient counseling, treatment eligibility, and long-term management.
